# High burden of drug therapy in adult congenital heart disease: polypharmacy as marker of morbidity and mortality

**DOI:** 10.1093/ehjcvp/pvz014

**Published:** 2019-03-23

**Authors:** Odilia I Woudstra, Joey M Kuijpers, Folkert J Meijboom, Marco C Post, Monique R M Jongbloed, Anthonie L Duijnhouwer, Arie P J van Dijk, Joost P van Melle, Thelma C Konings, Aeilko H Zwinderman, Barbara J M Mulder, Berto J Bouma

**Affiliations:** 1 Department of Cardiology, Heart Center, Amsterdam UMC, University of Amsterdam, Amsterdam Cardiovascular Sciences, Meibergdreef 9, AZ Amsterdam, The Netherlands; 2 Department of Cardiology, University Medical Center Utrecht, Heidelberglaan 100, CX Utrecht, The Netherlands; 3 Netherlands Heart Institute, Moreelsepark 1, EP Utrecht, The Netherlands; 4 Department of Cardiology, St. Antonius Hospital, Koekoekslaan 1, CM Nieuwegein, The Netherlands; 5 Department of Anatomy & Embryology, Leiden University Medical Center, Albinusdreef 2, ZA Leiden, The Netherlands; 6 Department of Cardiology, Leiden University Medical Center, Albinusdreef 2, ZA Leiden, The Netherlands; 7 Department of Cardiology, Radboud University Medical Center, Geert Grooteplein Zuid 10, GA Nijmegen, The Netherlands; 8 Department of Cardiology, University Medical Center Groningen, Hanzeplein 1, EZ Groningen, The Netherlands; 9 Department of Cardiology, VU University Medical Center, De Boelelaan 1117, HV Amsterdam, The Netherlands; 10 Department of Clinical Epidemiology, Biostatistics and Bioinformatics, Amsterdam UMC, University of Amsterdam, Meibergdreef 9, AZ Amsterdam, The Netherlands

**Keywords:** Adult congenital heart disease, Multiple medications, Polypharmacy, Dispensed drugs, Phenomapping, Cluster analysis, Adverse drug events

## Abstract

**Aims:**

To assess medication use in adult congenital heart disease (ACHD) patients compared to the age- and sex-matched general population, identify patterns of pharmacotherapy, and analyse associations between pharmacotherapy and adverse outcomes in ACHD.

**Methods and results:**

Data of 14 138 ACHD patients from the CONCOR registry [35 (24–48) years, 49% male] and age- and sex-matched referents (1:10 ratio) were extracted from the Dutch Dispensed Drug Register for the years 2006–14. Adult congenital heart disease patients had more cardiovascular and non-cardiovascular drugs than referents (median 3 vs. 1, *P* < 0.001). Polypharmacy, defined as ≥5 dispensed drug types yearly, was present in 30% of ACHD and 15% of referents {odds ratio [OR] = 2.47 [95% confidence interval (CI) 2.39–2.54]}. Polypharmacy was independently associated with female sex [OR = 1.92 (95% CI 1.88–1.96)], older age [for men: OR = 2.3/10 years (95% CI 2.2–2.4) and for women: OR = 1.6/10 years (95% CI 1.5–1.6); *P*_interaction_ < 0.001], and ACHD severity [mild: OR = 2.51 (95% CI 2.40–2.61), moderate: OR = 3.22 (95% CI 3.06–3.40), severe: OR = 4.87 (95% CI 4.41–5.38)]. Cluster analysis identified three subgroups with distinct medication patterns; a *low medication use* group (8-year cumulative survival: 98%), and a *cardiovascular* and *comorbidity* group with lower survival (92% and 95%, respectively). Cox regression revealed a strong association between polypharmacy and mortality [hazard ratio (HR) = 3.94 (95% CI 3.22–4.81)], corrected for age, sex, and defect severity. Polypharmacy also increased the risk of hospitalization for adverse drug events [HR = 4.58 (95% CI 2.04–10.29)].

**Conclusion:**

Both cardiovascular and non-cardiovascular medication use is high in ACHD with twice as much polypharmacy compared with the matched general population. Patients with polypharmacy had a four-fold increased risk of mortality and adverse drug events. Recognition of distinct medication patterns can help identify patients at highest risk. Drug regimens need repeating evaluation to assess the appropriateness of all prescriptions. More high-quality studies are needed to improve ACHD care with more evidence-based pharmacotherapy.

## Introduction

The adult congenital heart disease (ACHD) population is still growing and aging.^1^^,^^2^ Healthcare utilization is high, and drugs are more often prescribed in ACHD than in controls.^3^^,^^4^ Unlike other cardiovascular areas, evidence for drug therapy in ACHD is based on scarce clinical data and remains mostly empiric.^5^ Whether current pharmacological practice is efficient and safe in the long-term therefore remains questionable, but needs to be elucidated as drug therapy is increasingly used to address late complications. Pharmacological treatment in ACHD may start at a young age and may cumulate into chronic use of multiple medications. In elderly, it is known that the concurrent use of multiple medications, polypharmacy, is common (∼50%)^6^ and it is generally accepted that increased drug therapy is associated with adverse outcomes, such as adverse drug events (ADEs), hospitalizations, and death.^7^ However, data on polypharmacy in ACHD are lacking. Therefore, this study assessed medication use and polypharmacy in ACHD in comparison to the age- and sex-matched general population. Furthermore, we aimed to identify patterns of medication use in ACHD and to analyse the association between polypharmacy and adverse outcomes in ACHD.

## Methods

### Study population and data collection

This cohort study linked data of patients from the CONCOR registry,^8^ which includes adults (≥18 years) with congenital heart disease (CHD), to the national Dispensed Drug Register (DDR) of Statistics Netherlands (www.cbs.nl). For all Dutch residents, the DDR contains all dispensed outpatient drugs reimbursed by the compulsory basic Dutch health insurance. Drugs are classified following the Anatomical Therapeutic Chemical (ATC) classification (Supplementary material online, *Table S1*), which classifies drugs at five levels according to the organ/system on which they act (1st) and their therapeutic (2nd), pharmacological (3rd), and chemical properties (4th and 5th level).^9^ In the DDR, drugs are aggregated per person per year at the 3rd level of the ATC classification. Thus, specific drugs and their duration, timing, and daily doses within this 1-year window cannot be extracted. Receiving a specific drug is coded as dichotomous value for a full year, regardless of the amount of drugs dispensed. We, therefore, defined polypharmacy using the cumulative concept^10^ as ≥5 different drug types per calendar year, at the therapeutic (2nd) level of the ATC classification, to correct for changes in pharmacological classes.

Patients were matched with randomly selected age- and sex-matched reference subjects from the general population (1:10 ratio) to gain insight in the increase in medication use in ACHD compared to normal for these generally young persons (for details, see Supplementary material online, Methods and *Figure S1*). Subjects were followed from 2006 or CONCOR-inclusion until 2014 or death, using survival data from the national Cause of Death Register (CDR), which includes International Classification of Diseases (ICD) 10th revision coded causes of all deaths in Dutch citizens. From CONCOR, we obtained date of birth, inclusion date, sex, and main CHD, classified into mild, moderate, and severe CHD according to a much used consensus-based classification where proposed level of care and survival prospects differ per severity (Supplementary material online, *Table S2*).^11^^,^^12^

Additionally, data on hospitalizations for ADEs were collected via the Dutch Hospital Discharge Register (HDR) for the years 2006–12. The HDR contains person-linked discharge records of Dutch hospital admissions, including ICD-9 coded diagnoses and dates of admission. We defined hospitalizations for ADEs as admissions with ICD-9 codes 960–979 (poisoning by drugs, medicinal, and biological substances) as main diagnosis. The CDR was subsequently reviewed for ADEs as cause of death in all patients (ICD-10 codes T36–T50).

CONCOR was approved by the ethics boards of all participating centres^8^ and complies with the declaration of Helsinki.

### Statistical analysis

Statistical analyses were performed using RStudio V.1.0.153 (RStudio Team, Boston, MA, USA) and SPSS V.22 (IBM, Armonk, NY, USA). Data are summarized as *n* (%), mean ± standard deviation, and median [interquartile range (IQR)]. Two-sided *P*-values of <0.05 were considered statistically significant.

Drug use was described as percentage of years with dispensed drugs during the studied period. Generalized estimating equations with exchangeable working correlation and robust variance estimators were used to calculate odds ratios (ORs) for specific drugs and polypharmacy during the study in patients vs. matched referents, to determine whether sex, age, and CHD severity were independently associated with the presence of polypharmacy, and to plot predicted probability of polypharmacy by age in subsets per CHD severity. We performed subgroup analyses based on CHD type, sex, and age. A sensitivity analysis excluding sex hormones was performed to analyse the influence of oral contraceptives on the difference in polypharmacy between the sexes. We also performed sensitivity analysis excluding non-chronic drug types (including antibiotics, full list in Supplementary material online, *Table S3*) to test whether the cumulative definition of polypharmacy represented concurrent and continuous medication well.

To identify subgroups of patients with distinct patterns of medication relating to diseases of different organ systems, we used an unbiased machine learning approach. Of each patient, we determined whether drugs of the different anatomical classes of the ATC classification (1st level, Supplementary material online, *Table S1*) were used at year of inclusion. Hierarchical clustering was performed with the hclust and heatmap functions in R, using binary distance to calculate the dissimilarity matrix. The optimal number of clusters was estimated by maximizing the gap statistic using the gap method.^13^ Differences between clusters were compared using the χ^2^ and analysis of variance tests. Survival was assessed using the Kaplan–Meier analysis and compared between clusters using Cox hazard regression, adjusted for age, sex, and CHD severity.

For survival analyses, we excluded patients who were included in 2014 or died in their year of inclusion, because the yearly aggregated data required follow-up starting the following year. Cumulative survival for patients with and without polypharmacy at inclusion was assessed per CHD using the Kaplan–Meier curves. Associations between polypharmacy and all-cause mortality were analysed using multivariable Cox regression adjusted for age, sex, and CHD severity, with polypharmacy as time-varying factor. Interaction terms were used to analyse differences between CHD severities, and between ACHD patients and referents. Similarly, Cox hazards regression was used to analyse whether polypharmacy was associated with hospitalizations for ADEs in ACHD patients.

## Results

In total, 14 138 ACHD patients [age 35 (24–48) years, 49% male, 34% moderate, and 9% severe CHD] were followed for 8 (5–9) years (baseline characteristics in Supplementary material online, *Table S4*). Overall, 96 835 person-years of patients and 982 563 person-years of referents were analysed.

### Common drugs

*Table 1* shows the most commonly dispensed drugs. Adult congenital heart disease patients had higher use of cardiovascular drugs than referents, with highest use of antithrombotics {27 vs. 6% in referents, OR = 5.83 [95% confidence interval (CI) 5.60–6.07]}, β-blockers [24 vs. 6%, OR = 4.43 (95% CI 4.26–4.61)], and renin–angiotensin–aldosterone system (RAAS) inhibitors [21 vs. 7%, OR = 3.32 (95% CI 3.17–3.47)] (*Table **1**A*).

**Table 1:** Dispensed drugs

	ACHD patients, *n* person-years = 96 835 (%)	Matched referents, *n* person-years = 982 563 (%)	OR (95% CI)
**A. Cardiovascular drugs**			
Antithrombotics^a^ (e.g. vitamin K antagonists, NOACs, platelet aggregation inhibitors)	26.5	5.4	5.83 (5.60–6.07)
β-blockers^a^	23.7	6.3	4.43 (4.26–4.61)
RAAS inhibitors^a^	21.2	6.9	3.32 (3.17–3.47)
Diuretics^a^	11.4	3.8	3.23 (3.07–3.40)
Lipid modifiers^a^ (e.g. statins)	10.3	6.7	1.48 (1.39–1.56)
Calcium channel blockers^a^	6.1	2.6	2.17 (2.03–2.33)
Antiarrhythmics^a^	5.8	0.4	12.30 (11.23–13.47)
Other antihypertensives^a^	1.4	0.3	5.95 (5.14–6.90)
Antihaemorrhagics^a^ (e.g. vitamin K, coagulation factors)	1.0	0.2	6.30 (5.61–7.05)
Cardiac vasodilators^a^ (e.g. nitrates)	0.3	0.2	1.72 (1.31–2.24)
**B. Non-cardiovascular drugs used in >10% of ACHD**			
Systemic antibiotics^a^	37.8	19.7	2.45 (2.40–2.51)
Anti-inflammatory and antirheumatic products (e.g. NSAIDs, excluding aspirin)	17.3	17.3	1.01 (0.98–1.03)
Drugs for acid-related disorders^a^ (e.g. PPIs and antacids)	15.1	10.3	1.60 (1.54–1.66)
Dermatological corticosteroids^a^	13.6	10.6	1.33 (1.29–1.37)
Sex hormones^a^ (e.g. oral hormonal contraceptives)	11.2	8.6	1.33 (1.27–1.38)
Drugs for obstructive airway diseases^a^ [includes inhalants (adrenergics, corticosteroids) and systemic adrenergics]	10.3	6.9	1.57 (1.50–1.65)
Analgesics^a^ (e.g. opioids, aspirin)	10.2	6.7	1.58 (1.52–1.65)
Ophtalmologicals^a^ (topical ocular drugs)	10.2	7.5	1.40 (1.35–1.46)

Use of cardiovascular medication (A) and the most common non-cardiovascular medication (B) in ACHD patients compared with the use in matched referents from the general population. Drugs are presented according to the therapeutic classes of the Anatomical Therapeutic Chemical classification (Supplementary material online, *Table S1*).

ACHD, adult congenital heart disease; NOAC, non-vitamin K antagonist oral anticoagulant; RAAS, renin–angiotensin–aldosterone system.

a Significant at the *P*-value <0.001 level.

Remarkably, most non-cardiovascular drugs were also used more frequently in ACHD, especially systemic antibiotics [38 vs. 20%, OR = 2.45 (95% CI 2.40–2.51)], drugs for acid-related disorders [15 vs. 10%, OR = 1.60 (95% CI 1.54–1.66)] and drugs for obstructive airway disease [10 vs. 7% OR = 1.57 (95% CI 1.50–1.65)] (*Table **1**B*). Patients more commonly used drugs for thyroid disease than referents [3.8 vs. 2.0%, OR = 1.83 (95% CI 1.66–2.01)], especially patients with complete atrioventricular septal defects [OR = 15.69 (95% CI 9.53–25.83)] who often had Down syndrome [142 of 214 patients (67%)]. Antiepileptics also were more common [2.8 vs. 1.5%, OR = 1.84 (95% CI 1.68–2.02)], particularly in patients with transposition of the great arteries [OR = 4.58 (95% CI 2.87–7.33)] or a functionally univentricular heart [UVH; OR = 4.52 (95% CI 2.21–9.22)].

### Polypharmacy

**Figure 1:**
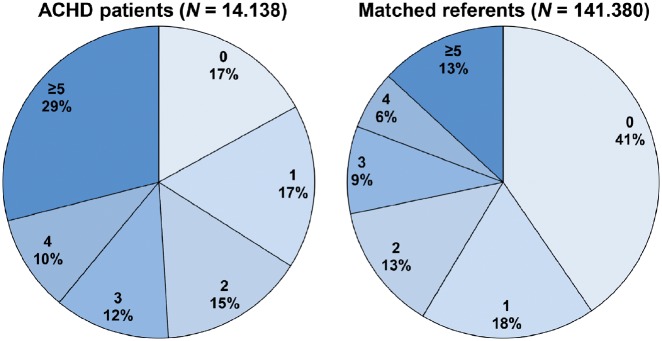
Amount of different drugs types at inclusion in adult congenital heart disease patients and matched referents.

Adult congenital heart disease patients had a median of three different dispensed drugs at year of inclusion compared to a median of one in reference subjects (*P* < 0.001) (*Figure 1*). Twice as little patients were free of dispensed drugs at inclusion compared to referents (17 vs. 40%, *P* < 0.001) (most common drugs in polypharmacy: Supplementary material online, *Table S5*).

**Figure 2:**
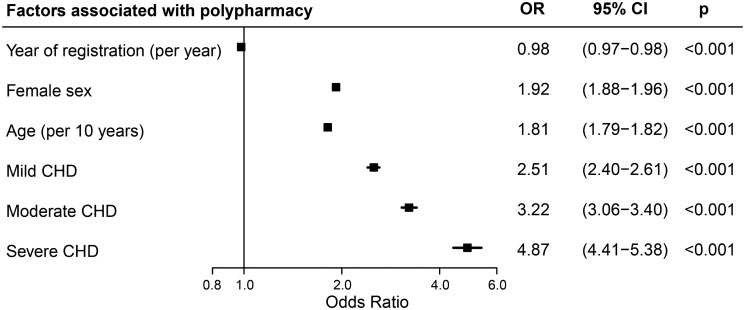
Factors independently associated with polypharmacy in the entire cohort, showing odds ratios (OR) for polypharmacy during the study period. CHD, congenital heart defect.

Mean prevalence of polypharmacy during the study was 30% in ACHD compared to 15% in referents [OR = 2.47 (95% CI 2.39–2.54)]. Polypharmacy was independently associated with older age, female sex, and CHD severity [mild: OR = 2.51 (95% CI 2.40–2.61), moderate: OR = 3.22 (95% CI 3.06–3.40), and severe: OR = 4.87 (95% CI 4.41–5.38)] (*Figure 2*). It was particularly present in patients with a UVH [44%, OR = 8.54 (6.62–11.02)], with many cardiovascular drugs indicating high-cardiac morbidity, and in patients with the Marfan syndrome [45%, OR = 4.60 (95% CI 3.98–5.31)], with notable use of cardiovascular drugs, ocular medication [18%, OR = 2.61 (95% CI 2.20–3.11)], and analgesics [16%, OR = 2.55 (95% CI 2.16–3.01)], reflecting ocular and skeletal problems (e.g. scoliosis) often seen in these syndromic patients.

**Figure 3:**
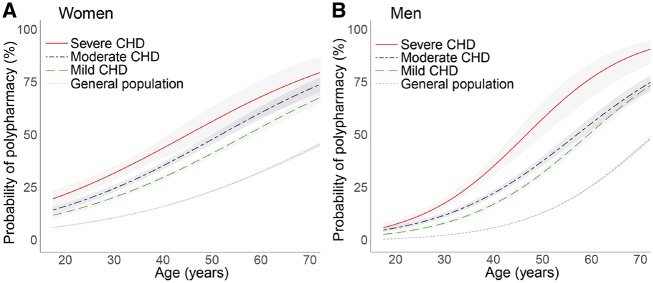
Probability of polypharmacy for women (*A*) and men (*B*) by age, stratified for congenital heart defect severity, and compared with age- and sex-matched referents.

Even in mild CHD, polypharmacy was already as common in 45-year-old female and 50-year-old male patients as in 65-year-old persons from the general population (*Figure 3*). Already 48% of patients with severe CHD had polypharmacy at the age of 45 years, a proportion only seen for persons aged ≥70 years in the general population.

Overall, polypharmacy was more common in women than men [OR = 1.92 (95% CI 1.88–1.96)]. It was already present in 24% of female patients under 40 years (vs. 12% of female referents <40), with high use of antibiotics (41%) and sex hormones including contraceptives (31%). Even after exclusion of sex hormones, polypharmacy prevalence remained higher in women [OR = 1.88 (95% CI 1.74–1.78)]. In men, polypharmacy was less common at young age but showed a steep incline with age [OR = 2.3/10 years (95% CI 2.2–2.4), for women: OR = 1.6/10 years (95% CI 1.5–1.6); *P*_interaction_ < 0.001]; 40% of male patients over 40 years had polypharmacy (vs. 19% of male referents >40), with high use of antithrombotics (46%) and RAAS inhibitors (23%). These sex- and age-specific differences were seen both in patients and referents.

Mean prevalence of polypharmacy was still 25% in ACHD compared to 12% in matched referents [OR = 2.39 (95% CI 2.32–2.48)] when non-therapeutic and non-chronic drugs were excluded for sensitivity analysis.

### Patterns of medication use

**Figure 4:**
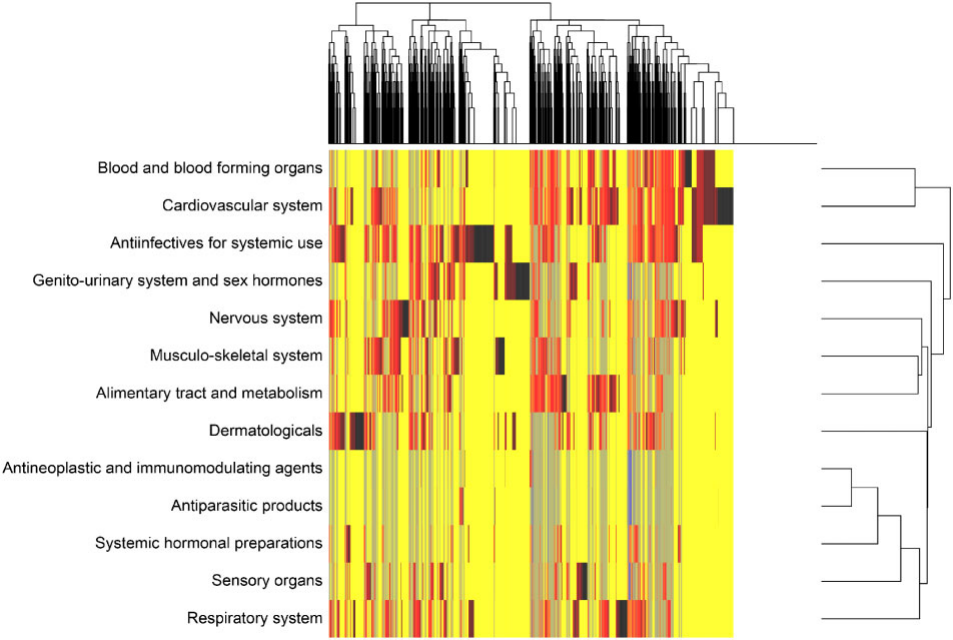
Medication phenotype heat map of adults with congenital heart disease. *Columns* represent individual patients and *rows* represent independent phenotypes of dispensed drugs aggregated at the anatomical level of the Anatomical Therapeutic Chemical classification. Red indicates increased value, yellow intermediate, and blue decreased value of a drug. White columns represent 2409 patients with zero drugs.

The phenotype heat map created by hierarchical clustering of medication used in ACHD demonstrated heterogeneity among patients (*Figure 4*). The use of drugs acting on the cardiovascular and blood & blood forming organs (mainly antithrombotics) seemed to co-occur most.

**Figure 5:**
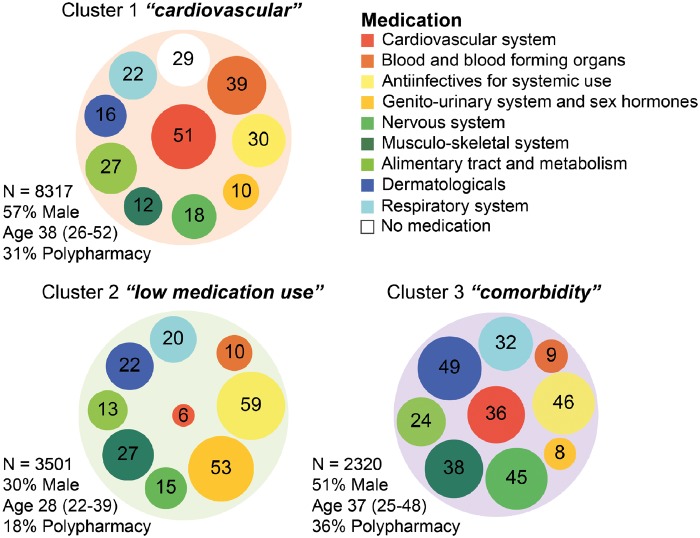
Clinical characteristics and medication use at inclusion stratified by phenogroup. Numbers represent the percentage of patients per subgroup with medication for the different organ systems used at year of inclusion.

The analysis arrived at three clusters as the optimal number to reflect phenotypic variability (Supplementary material online, *Figure S2*). The clusters differed significantly (Supplementary material online, *Table S6*). As shown in *Figure 5*, Cluster 1 (*n* = 8317) had the highest proportion of patients with drugs acting on the cardiovascular and blood & blood forming systems. This *cardiovascular* cluster was the oldest and had most patients with severe CHD (10%) and left sided lesions (e.g. bicuspid aortic valve: 11%). Cluster 2 (*n* = 3501) mainly contained patients using anti-infectives and genito-urinary medication (sex hormones), but relative low use of other drugs, with polypharmacy in only 18% of patients. This *low medication use* cluster contained young, mainly female (70%) patients, mostly with mild defects (61%). In Cluster 3 (*n* = 2320), the *comorbidity* cluster, many patients used extra-cardiac medication. It had the highest proportion of patients with polypharmacy (36%) and genetic syndromes (7%).

After 8 years of follow-up, cumulative survival was 92% in the *cardiovascular* cluster, 98% in the *low medication use* cluster, and 95% in the *comorbidity* cluster. Corrected for age, sex, and CHD severity, survival was better for the *low medication use* vs. *cardiovascular* cluster [hazard ratio (HR) = 0.50 (95% CI 0.37–0.78), *P* < 0.001], but, despite the distinct medication patterns, did not differ between the *comorbidity* and *cardiovascular* cluster [HR = 0.89 (95% CI 0.71–1.11), *P* = 0.31].

### Polypharmacy and outcome

**Figure 6:**
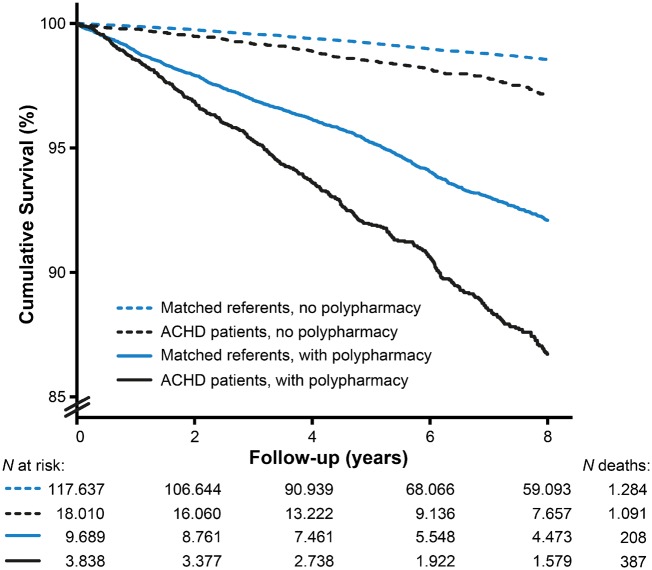
Kaplan–Meier survival curve of adult congenital heart disease (ACHD) patients and matched referents with and without polypharmacy at inclusion.

**Figure 7:**
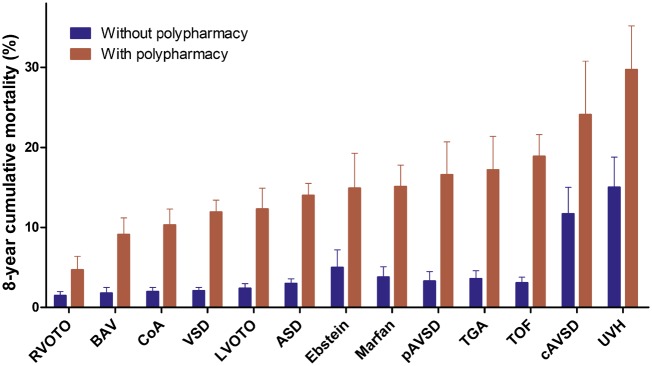
Eight-year cumulative mortality for patients with and without polypharmacy at inclusion per congenital heart defect. ASD, atrial septal defect; BAV, bicuspid aortic valve; cAVSD, complete atrioventricular septal defect; CoA, coarctation of the aorta; Ebstein, Ebstein’s anomaly; LVOTO, left ventricular outflow tract obstruction; pAVSD, partial atrioventricular septal defect; RVOTO, right ventricular outflow tract obstruction; TGA, transposition of the great arteries; TOF, tetralogy of Fallot; UVH, univentricular heart; VSD, ventricular septal defect.

Survival analyses included 13 527 patients and 135 647 referents. During 7 (5–8) years, 595 (4%) patients and 2375 (2%) referents died (*Figure 6*). Eight-year mortality was higher in patients with polypharmacy at inclusion compared to those without polypharmacy (*Figure 7*). Corrected for age, sex, and defect severity, polypharmacy during the study was strongly associated with all-cause mortality in ACHD [HR = 3.94 (95% CI 3.22–4.81)]. The age- and sex-adjusted association was similar between the CHD severities (*P*_interaction_ = 0.96 for moderate and *P*_interaction_ = 0.70 for severe CHD compared to mild CHD) and was significantly stronger in ACHD patients than in referents (*P*_interaction_ < 0.001).

A total of 10 015 ACHD patients were uniquely identified in the HDR between 2005 and 2012. During a median of 5 (IQR 3–6) years, 21 ACHD patients were hospitalized for an ADE. Increasing drug amounts were associated with ADEs [HR = 1.20/dispensed drug (95% CI 1.10–1.32)]. Patients with polypharmacy were at markedly higher risk of hospitalization for an ADE compared to patients without polypharmacy [HR = 4.03 (95% CI 1.67–9.73)]. None of the patients that died during the study had ADEs as cause of death.

## Discussion

This study shows that ACHD patients not only use more cardiovascular medication than the general population, but also use more extra-cardiac drugs, cumulating into polypharmacy in 30% of the patients compared to only 15% of referents. The study identified distinct medication patterns, which differed by age, sex, and CHD. Furthermore, patients with polypharmacy had an almost four-fold higher risk of all-cause mortality and almost five-fold higher risk of hospitalizations for ADEs.

Recently, ACHD investigators have stressed the need for more evidence regarding drug therapy in this growing population.^5^ Trials investigating safety and efficacy of drugs in ACHD often remain small.^14^^,^^15^ The existing pool of evidence in this area therefore only grows slowly and remains largely empiric. Some epidemiologic studies have identified common drugs in ACHD cohorts.^4^^,^^16^ However, this study is the first to investigate polypharmacy and its associations with clinical characteristics and outcome in ACHD. Furthermore, this is the largest study comparing medication use in ACHD to the general population.

Previous studies focusing on other chronic conditions, such as diabetes mellitus, chronic kidney disease, and chronic heart failure, have shown comparably high odds for polypharmacy of these diseases.^17^^,^^18^ Compared to these populations, ACHD patients are special due to their young age and lifelong disease which may involve both cardiac and extra-cardiac comorbidities. Polypharmacy in 15% of the age-matched referents may seem high but is close to other findings using cumulative definitions of polypharmacy during a 1-year period.^19^ Not surprisingly, polypharmacy risk in our study increased with increasing CHD severity, which involves more cardiovascular complications requiring medical intervention.^16^^,^^20^

Apart from common use of cardiovascular drugs, use of many non-cardiovascular drugs was increased in ACHD. Previous research showed increased prevalence of drugs related to asthma and epilepsy in patients who underwent surgery for a CHD as children.^4^ Especially in patients with genetic syndromes, extra-cardiac comorbidities are common.^4^^,^^21^ In our cohort, we saw increased use of a large range of drugs, including drugs for acid-related disorders, dermatologicals, and sex hormones. This indicates high prevalence of extra-cardiac comorbidities in the ACHD population. Contra-indications for pregnancy are more common in women with cardiovascular disease^22^ and may explain a higher preventive use of oral contraceptives in ACHD.

Interestingly, polypharmacy was even increased in mild CHD and at young age, reflecting decreased health even in these mildly affected patients. Alternatively, the increase in medication use may originate from intensive surveillance that facilitates early diagnosis and treatment.^3^ The particularly higher prevalence of polypharmacy in female compared to male ACHD patients at young age is in line with general sex differences that depend on differences including prevalence of morbidities and adverse drug effects, need for anticonceptives, and a lower likelihood to seek preventive healthcare in men.^23^

Cluster analysis revealed three distinct patterns of medication use in ACHD, described as *cardiovascular*, *low medication use*, and *comorbidity* patterns. Cluster analysis based on phenotypical data has been used previously to identify distinct subgroups within other heterogeneous populations.^24^^,^^25^ This unbiased approach makes it possible to identify patterns regardless of assumptions about clinical correlations. The identification of such distinct subgroups could be used to help target therapies and trials in heterogeneous syndromes such as ACHD. Clinical trials are prone to select patients without marked comorbidity, but concurrent use of different drugs is important to identify due to increased risk of drug–drug interactions and ADEs.^26^^,^^27^ This may be most crucial in the *comorbidity* subgroup.

This study showed, without implying causality, that patients with polypharmacy had a four-fold higher mortality risk (HR = 3.94), independent of age, sex, and defect severity. Furthermore, risk of hospitalization for adverse drug events was nearly five times higher in patients with polypharmacy (HR = 4.58). Interestingly, polypharmacy in the ACHD population was more associated with mortality than in the general population. Patients with polypharmacy may be sicker (needing therapy) than referents with polypharmacy (who e.g. often have statins as prevention). Whether an increased amount of drugs is an independent risk factor or a mere measure of poor health and multimorbidity, remains to be elucidated.^6^^,^^7^ Polypharmacy may enhance risk of adverse drug events, including bleeding due to antithrombotics,^28^ and increased amounts of drugs correlate with hospitalizations for adverse drug reactions.^26^^,^^27^ Notably, drugs often prescribed in ACHD, especially anticoagulants, are among the drugs most commonly causing ADE-related emergency department visits and hospitalizations.^29^^,^^30^ Benefits of prescribing may outweigh the risks of ADEs, but evidence of beneficial effects of many therapies in ACHD is still limited.^5^ In elderly, guidelines with criteria to start and stop certain drugs have been established to minimize inappropriate prescribing,^31^ and it has been suggested that deprescribing to reduce inappropriate polypharmacy can reduce mortality without harm.^32^^,^^33^

### Clinical implications

The remarkably high prevalence of polypharmacy in ACHD shows that experience with managing polypharmacy is needed in the efficient management of these patients. Physicians should carefully judge drug indications in ACHD, especially as pharmacotherapy is often based on low-level evidence extrapolated from non-ACHD studies or small studies involving heterogeneous ACHD patients. Long-term use of some medication, e.g. amiodarone, may be suboptimal due to side effects.^5^ Occasionally, withdrawal of longstanding therapy with only weak indications might be an option. Trials that examine efficacy and safety of drug therapy in ACHD are warranted, and the effects of longstanding polypharmacy in these patients need to be studied further to enhance guidelines on the management of this complex population.

### Methodological issues

These data from national administrative databases enable insightful comparisons with the general population. Automated data collection limits recall bias seen in questionnaires and data on dispensed drugs provide more accurate information on actual drug consumption than medical records, as these prescriptions have been filled. However, actual drug consumption may be overestimated, as we have no data on compliance. Non-compliance is of importance because it is associated with mortality and increases with treatment intensity and duration,^34^^,^^35^ although compliance in the Netherlands is reported to be high (>80%).^36^

The lack of clinical detail inherent to administrative data introduces indication bias, as no information on drug indications, comorbidities, and functional status are available. We used the consensus-based severity classification to subdivide patients with different risks. However, mortality risk may vary within specific CHDs due to late complications, such as pulmonary hypertension in patients with septal defects. Therefore, these data do not provide information about individual patients, but give insight on a population level. Furthermore, appropriateness of polypharmacy is not assessed and associations with mortality have to be interpreted with caution, as polypharmacy may mark high-risk patients with multimorbidity.

Other limitations inherent to the data set include unavailability of data on over-the-counter medication, and data on treatment duration, daily doses, and specific distributed drugs. Our cumulative measures of polypharmacy may overestimate the prevalence of simultaneous pharmacotherapy, due to inclusion of successive and non-chronic drugs in the observed time frame. We limited this by aggregating drugs by therapeutic class, correcting for switches in pharmacological class. Such cumulative definitions of polypharmacy are common and give comparable, clinically relevant, and as reliable results as other measures of polypharmacy.^10^^,^^19^^,^^37^

## Conclusion

In conclusion, ACHD patients used both more cardiovascular and non-cardiovascular medication compared to the general population, with polypharmacy in 30% of ACHD vs. just 15% of referents. Polypharmacy was even common in mild CHD at young ages. We identified different medication patterns that could be taken into account to help target therapies and trials in this heterogeneous population. As patients with polypharmacy had a four-fold higher risk of death and adverse drug events, daily clinical care of ACHD patients must include regular evaluation of their medication regimen, particularly in case of polypharmacy. Further clinical trials to investigate risks and benefits of pharmacotherapy remain needed to come to more evidence-based treatment in this population.

## Supplementary Material

pvz014_Supplementary_Data
