# Supplementary material for: High burden of drug therapy in adult congenital heart disease: polypharmacy as marker of morbidity and mortality
Source: Eur Heart J Cardiovasc Pharmacother. 2019 Mar 23;5(4):216–25. doi: 10.1093/ehjcvp/pvz014 (PMC6749841; doi:10.1093/ehjcvp/pvz014)
Supplement: pvz014_Supplementary_Data [file pvz014_supplementary_data.pdf]

SUPPLEMENTARY MATERIAL

High burden of drug therapy in adult congenital heart disease: polypharmacy as marker of morbidity and mortality

Odilia I. Woudstra MD[1,2], Joey M. Kuijpers MD[1,3], Folkert J. Meijboom MD PhD[2], Marco C. Post MD PhD[4], Monique R.M. Jongbloed MD PhD[5,6], Anthonie L. Duijnhouwer MD[7], Arie P.J. van Dijk MD PhD[7], Joost P. van Melle MD PhD[8], Thelma C. Konings MD[9], Aeilko H. Zwinderman PhD[10], Barbara J.M. Mulder MD PhD[1,3], Berto J. Bouma MD PhD[1]

1. Heart Center; Department of Cardiology, Amsterdam UMC, University of Amsterdam, Amsterdam Cardiovascular Sciences, Meibergdreef 9, 1105 AZ, Amsterdam, The Netherlands
2. Department of Cardiology, University Medical Center Utrecht, Heidelberglaan 100, 3584 CX, Utrecht, The Netherlands
3. Netherlands Heart Institute, Moreelsepark 1, 3511 EP, Utrecht, The Netherlands
4. Department of Cardiology, St. Antonius Hospital, Koekoekslaan 1, 3435 CM, Nieuwegein, The Netherlands
5. Department of Anatomy & Embryology, Leiden University Medical Center, Albinusdreef 2, 2333 ZA, Leiden, The Netherlands
6. Department of Cardiology, Leiden University Medical Center, Albinusdreef 2, 2333 ZA, Leiden, The Netherlands
7. Department of Cardiology, Radboud University Medical Center, Geert Grooteplein Zuid 10, 6525 GA, Nijmegen, The Netherlands
8. Department of Cardiology, University Medical Center Groningen, Hanzeplein 1, 9713 EZ, Groningen, The Netherlands
9. Department of Cardiology, VU University Medical Center, De Boelelaan 1117, 1081 HV, Amsterdam, The Netherlands
10. Department of Clinical Epidemiology, Biostatistics and Bioinformatics, Amsterdam UMC, University of Amsterdam, Meibergdreef 9, 1105 AZ, Amsterdam, The Netherlands

TABLE OF CONTENTS

Supplementary table 1	p. 3
Supplementary methods	p. 6
Supplementary figure 1	p. 7
Supplementary table 2	p. 8
Supplementary table 3	p. 10
Supplementary table 4	p. 11
Supplementary table 5	p. 12
Supplementary figure 2	p. 13
Supplementary table 6	p. 14
References	p. 15

Supplementary table 1: Coding of drug classes according to the Anatomical Therapeutic Chemical (ATC) classification system.

ATC code 1st	ATC code 2nd	Description	Includes the following (3rd or 4th level of ATC classification)
**A**		**Alimentary tract and metabolism**	
A	01	Stomatological preparations	Local oral treatments
A	02	Drugs for acid related disorders	Antacids and drugs for peptic ulcer disease (i.a. proton-pump inhibitors)
A	03	Drugs for functional gastrointestinal disorders	Antispasmodics, anticholinergics
A	04	Antiemetics & antinauseants	
A	05	Bile and liver therapy	
A	06	Drugs for constipation	
A	07	Antidiarrheals, intestinal antiinflammatory/antiinfective agents	
A	08	Antiobesity preparations excl. diet products	
A	09	Digestives, incl. enzymes	
A	10	Drugs used in diabetes	Insulines, blood glucose lowering drugs
A	11	Vitamins	
A	12	Mineral supplements	I.a. potassium, calcium
A	14	Anabolic agents for systemic use	
A	16	Other alimentary tract and metabolism products	Amino acids, enzymes
**B**		**Blood and blood forming organs**	
B	01	Antithrombotic agents	Platelet aggregation inhibitors, anticoagulants (vitamin K antagonists, non-vitamin K antagonists)
B	02	Antihemorrhagics	I.a. vitamin K, other coagulation factors
B	03	Antianemic preparations	Iron preparation, vitamin B12 and folic acid
B	05	Blood substitutes and perfusion solutions	
B	06	Other hematological agents	
**C**		**Cardiovascular system**	
C	01	Cardiac therapy	Cardiac glycosides (i.a. digitalis), antiarrhythmics, cardiac vasodilators (i.a. nitrates)
C	02	Antihypertensives	Antiadrenergic agents
C	03	Diuretics	Thiazides, sulfonamides, potassium-sparing diuretics
C	04	Peripheral vasodilators	
C	05	Vasoprotectors	Local treatment of hemorrhoids and varices
C	07	Beta blocking agents	
C	08	Calcium channel blockers	
C	09	Agents acting on renin-angiotensin system	ACE inhibitors, angiotensin II receptor blockers
C	10	Lipid modifying agents	I.a. Statins
**D**		**Dermatologicals**	
D	01	Antifungals for dermatological use	
D	02	Emollients and protectives	
D	03	Preparations for treatment of wounds and ulcers	
D	04	Antipruritics, incl. antihistamines, anaestetics, etc.	
D	05	Antipsoriatics	
D	06	Antibiotics and chemotherapeutics for dermatoligical use	
D	07	Corticosteroids, dermatiological preparations	
D	08	Antiseptics and disinfectants	
D	09	Medicated dressings	
D	10	Anti-acne preparations	
D	11	Other dermatological preparations	

ATC code 1st	2nd	Description	Includes the following (3rd or 4th level of ATC classification)
**G**		**Genito urinary system and sex hormones**	
G	01	Gynaecological antiinfectives and antiseptics	
G	02	Other gynaecologicals	Uterotonics, intrauterine contraceptives
G	03	Sex hormones and modulators of the genital system	Hormonal contraceptives, androgens, estrogens, progesterons
G	04	Urologicals	I.a. drugs used in prostatic hypertrophy
**H**		**Systemic hormonal preparations, excl. sex hormones and insulins**	
H	01	Pituary and hypothalamic hormones and analogues	
H	02	Corticosteroids for systemic use	
H	03	Thyroid therapy	Thyroid hormones, antithyroid drugs iodine therapy
H	04	Pancreatic hormones	Glucagon
H	05	Calcium homeostasis	Parathyroid hormones, anti-parathyroid drugs
**J**		**Antiinfectives for systemic use**	
J	01	Antibacterials for systemic use	
J	02	Antimycotics for systemic use	
J	04	Antimycobacterials	
J	05	Antivirals for systemic use	
J	06	Immune sera and immunoglobulins	
J	07	Vaccines	
**L**		**Antineoplastic and immunomodulating agents**	
L	01	Antineoplastic agents	
L	02	Endocrine therapy	
L	03	Immunostimulants	
L	04	Immunosuppressants	
**M**		**Musculo-skeletal system**	
M	01	Anti-inflammatory and antirheumatic products	Non-steroidal anti-inflammatory drugs (NSAIDs), excluding aspirin
M	02	Topical products for joint and muscular pain	
M	03	Muscle relaxants	
M	04	Antigout preparations	
M	05	Drugs for treatment of bone disease	Bisphosphonates
M	09	Other drugs for disorders of the musculo-skeletal system	
**N**		**Nervous system**	
N	01	Anesthetics	
N	02	Analgesics	Opioids, aspirin, antimigraine drugs
N	03	Antiepileptics	
N	04	Anti-parkinson drugs	
N	05	Psycholeptics	Antipsychotics, anxiolytics, hypnotics, sedatives
N	06	Psychoanaleptics	Antidepressants, psychostimulants, anti-dementia drugs
N	07	Other nervous system drugs	Parasympathicomimetics, drugs used in addictive disorders, antivertigo preparations
**P**		**Antiparasitic products, insecticides and repellents**	
P	01	Antiprotozoals	
P	02	Anthelmintics	
P	03	Ectoparasiticides, incl. scabicides, insecticides and repellents	

ATC code 1st	2nd	Description	Includes the following (3rd or 4th level of ATC classification)
**R**		**Respiratory system**	
R	01	Nasal preparations	Topical and systemic nasal decongestants
R	02	Throat preparations	Local antiseptics, antibiotics, anesthetics
R	03	Drugs for obstructive airway diseases	Inhalants (adrenergics, corticosteroids), systemic adrenergics
R	05	Cough and cold preparations	
R	06	Antihistamines for systemic use	
R	07	Other respiratory system products	
**S**		**Sensory organs**	
S	01	Ophtalmologicals	Local ocular drugs including anti-infectives, antiallergics, corticosteroids, antiglaucoma drugs
S	02	Otologicals	Ear drops including anti-infectives, corticosteroids, analgesics
S	03	Ophtalmological and otological preparations	
**V**		**Various**	
V	01	Allergens	
V	03	All other therapeutic products	I.a. drugs for treatment of hyperkalemia, hypoglycaemia, antidotes
V	04	Diagnostic agents	
V	06	General nutrients	
V	07	All other non-therapeutic products	I.a. plasters, diluting agents
V	08	Contrast media	
V	09	Diagnostic radiopharmaceuticals	
V	10	Therapeutic radiopharmaceuticals	
V	20	Surgical dressings	

First and second level of the Anatomical Therapeutic Chemical classification code.
Abbreviations: ACE, angiotensin converting enzyme; ATC, anatomical therapeutic chemical; i.a.; among others.

Supplementary methods:

Derivation of the full study cohort

CONCOR patients

For this study, CONCOR was linked to the Municipal Personal Records Database (MPRD) of Statistics Netherlands. The MPRD is a longitudinal registry containing all registered Dutch residents since 1995. CONCOR patients were identified in the MPRD by Statistics Netherlands using full postal code (4 digits, 2 letters), sex, and birthdate as the linkage key. Patients thus uniquely identifiable got an identification number, corresponding to unique persons in the Dispensed Drug Register (DDR) and Cause of Death Register (CDR). Data of the DDR and CDR were available for the years 2006-2014.

Supplementary figure 1 shows the derivation of the study cohort. Of 16,066 patients included in CONCOR at time of linkage, 378 (2.4%) had opted out of, or had no registered postal code for linkage to external registries; 851 (5.3%) were not unique on the MPRD linkage key. Of the 14,837 patients successfully linked to the MPRD, 119 had died before 2006 and 580 were included after 2014. The ACHD cohort for medication analyses therefore consisted of 14,138 patients. For survival analyses, an additional 611 patients were excluded (see Methods section in the manuscript).

Additionally, the Hospital Discharge Register (HDR) was used for analyses on hospitalizations for adverse drug events (ADE). Data of the HDR were available for the years 2007-2012. In the HDR, MPRD-unique persons may lose unicity on the linkage key at any time in the study-period, as the HDR is linked to the MPRD using 4-digit postal code, sex and birthdate. A total of 10,015 patients were included in analyses regarding hospitalizations for ADE.

Matched reference subjects

For every ACHD patient, ten sex- and birthyear-matched persons from the MPRD were selected to create an age- and sex-matched reference cohort from the general Dutch population (Supplementary figure 1). Exclusion criteria for referents included being included in the CONCOR-registry, having died before inclusion of the matched ACHD patient, and having emigrated during follow-up of the matched ACHD patient. For referents fulfilling any of the exclusion criteria, new referents were randomly selected from the MPRD until ten referents per patient were available.

Supplementary figure 1: Derivation of the ACHD and reference cohorts.

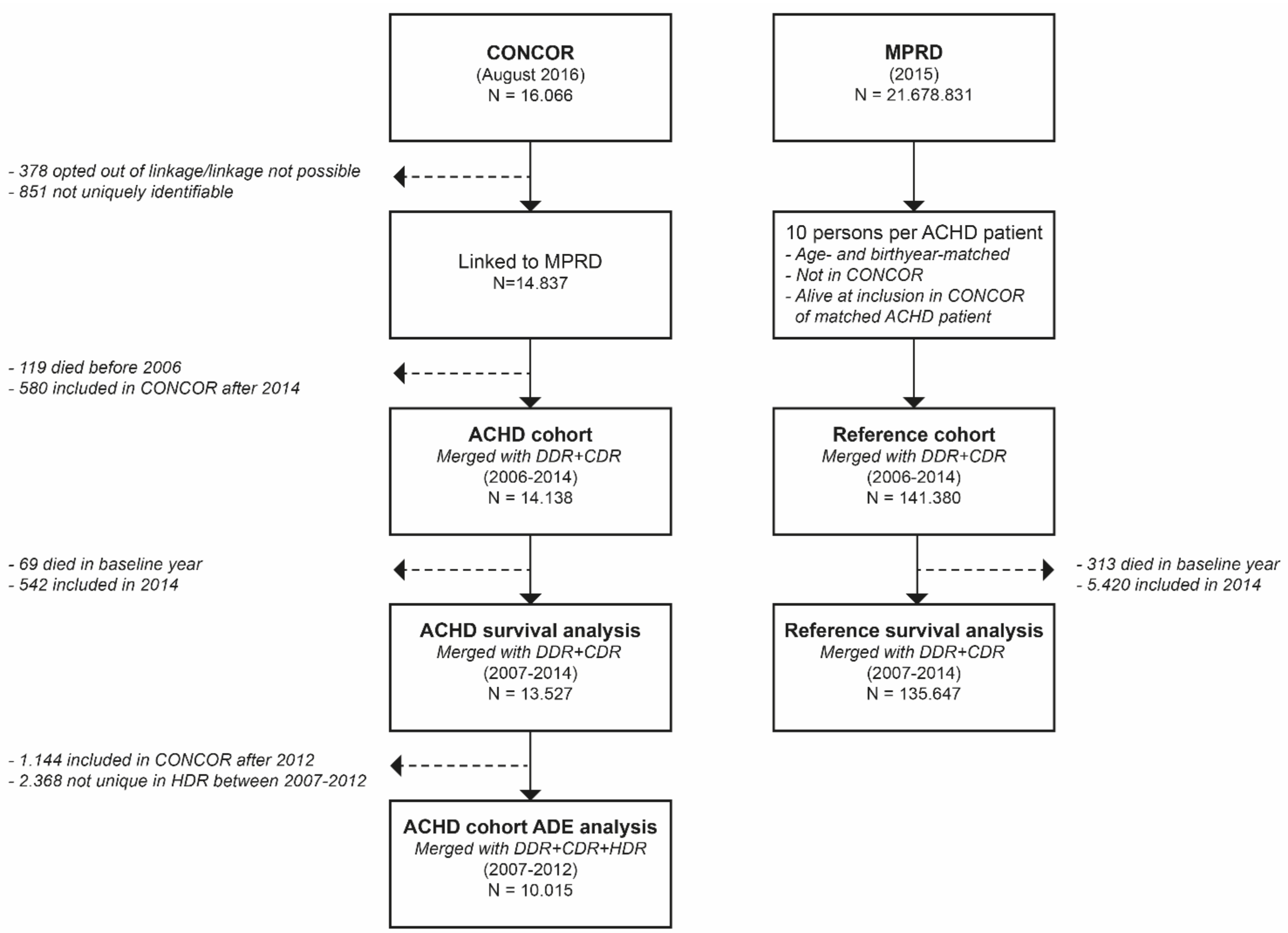

Abbreviations: ACHD, adult congenital heart disease; ADE, adverse drug event; CDR, Cause of Death Register; DDR, Dispensed Drug Register; HDR, Hospital Discharge Register; MPRD, Municipal Personal Records Database.

<u>Supplementary table 2:</u> Categorization of main congenital heart defect by severity.

Severity	Primary CHD lesion	Definition of EPCC code	EPCC code
**Severe CHD**			
	Functionally univentricular heart	Tricuspid atresia	06.01.01
		Functionally univentricular heart	01.01.22
		Double inlet LV	01.04.04
		Double inlet RV	01.04.03
	Transposition complex	Complete transposition of great arteries (IVS)	01.01.02
		Discordant VA connections (TGA)	01.05.01
		Congenitally corrected transposition of great arteries	01.01.03
	Double outlet right ventricle	Double outlet RV	01.01.04
		Double outlet RV - transposition type	01.01.18
		Double outlet RV - Fallot type	01.01.17
		Double outlet RV - with non-committed VSD	01.01.19
	Pulmonary atresia	Pulmonary atresia + VSD (including Fallot type)	01.01.06
		Pulmonary atresia + intact ventricular septum	01.01.07
		Pulmonary atresia + VSD + 'MAPCA'(s)	01.01.25
		Pulmonary atresia	09.05.11
	Other severe CHD	Common arterial trunk	09.01.01
		Total mirror imagery (atrial situs inversus)	03.01.03
**Moderate CHD**			
	AVSD	AVSD: isolated atrial component (primum ASD)	06.06.01
		AVSD: atrial & ventricular components (complete)	06.06.09
		Atrioventricular septal defect	06.06.00
		AVSD: isolated ventricular component	06.06.08
	Aortic valve/ LVOT lesion	Marfan syndrome	14.02.17
		Subaortic stenosis due to fibromuscular shelf	07.09.03
		Supravalvar aortic stenosis	09.16.00
		LV outflow tract obstruction	07.09.01
		Subaortic stenosis	07.09.00
		Ascending aorta abnormality	09.16.10
	Aortic coarctation	Aortic coarctation	09.29.01
		Interrupted aortic arch	09.29.31
		Aortic arch hypoplasia (tubular)	09.29.11
		Aortic arch abnormality	09.28.00
	Mitral lesion	True cleft of mitral valve	06.02.36
	Other left-sided CHD	Cor triatriatum (divided left atrium)	05.02.01
	ASD (non-secundum type)	Sinus venosus ASD	05.05.00
		Common atrium (virtual absence of atrial septum)	05.06.01
		Interatrial communication (ASD) through coronary sinus orifice	05.05.03
	VSD (multiple)	Multiple VSDs	07.15.04
	Aorto-pulmonary window	Aortopulmonary window	09.04.01
	Tetralogy of Fallot	Tetralogy of Fallot	01.01.01
		Absent pulmonary valve syndrome – Fallot type	09.05.25
	Pulmonary valve/ RVOT lesion (non-ToF)	RV outflow tract obstruction	07.05.01
		Supravalvar pulmonary trunk stenosis	09.07.13
		Pulmonary valvar abnormality	09.05.00
		Subpulmonary stenosis	07.05.30
	Ebstein's anomaly	Ebstein's malformation of tricuspid valve	06.01.34
	Other CHD	Double chambered RV	07.03.01
		Partially anomalous pulmonary venous connection(s)	04.07.01
		Totally anomalous pulmonary venous connection	04.08.05
		Coronary fistula	09.45.01
		Anomalous origin of coronary artery from pulmonary artery	09.41.01
		Partially anomalous pulmonary venous connections: Scimitar syndrome	01.01.16
		Coronary artery: anomalous aortic origin/course	09.42.00
		Coronary arterial abnormality	09.46.00
		Arteriovenous fistula	09.19.01
		Coronary sinus abnormality	04.04.00
		Pulmonary arteriovenous fistula	09.19.05

**Mild CHD**			
	Aortic valve/ LVOT lesion	Bicuspid aortic valve	09.15.22
		Aortic valvar stenosis - congenital	09.15.01
		Aortic regurgitation - congenital	09.15.07
		Aortic valvar abnormality	09.15.00
		Ascending aorta dilation	09.16.09
		Aortic valvar stenosis	09.15.13
	Mitral defect	Mitral valvar prolapse	06.02.35
		Mitral valvar stenosis - congenital	06.02.07
		Mitral regurgitation - congenital	06.02.25
		Mitral valvar abnormality	06.02.00
		Supravalvar mitral ring	05.02.02
	Other left-sided CHD	Double aortic arch	09.28.09
		Right aortic arch	09.28.15
		LA abnormality	05.02.00
	VSD	VSD	07.10.00
		Perimembranous VSD	07.10.01
		Muscular VSD	07.11.01
		Subarterial VSD	07.12.00
		Doubly committed subarterial VSD	07.12.01
		Inlet VSD	07.14.05
	ASD	ASD within oval fossa (secundum)	05.04.02
		ASD	05.04.01
	PDA	Patent arterial duct (PDA)	09.27.21
	Pulmonary valve/ RVOT lesion (non-ToF)	Pulmonary valvar stenosis - congenital	09.05.04
		Pulmonary stenosis	09.05.92
		Peripheral pulmonary arterial stenoses - at/beyond hilar bifurcation	09.10.06
		Pulmonary trunk (MPA) abnormality	09.07.00
		Pulmonary regurgitation - congenital	09.05.22
	Other right-sided CHD	Tricuspid valvar abnormality	06.01.00
		RA abnormality	05.01.00
		Tricuspid regurgitation - congenital	06.01.25
	Other mild CHD	Congenital complete heart block	11.06.16
		Dextrocardia: heart predominantly in right hemithorax	02.01.02
		Aneurysm of membranous septum	07.20.01
		Left SVC persisting to coronary sinus	04.01.01
		Superior caval vein abnormality	04.01.00

In CONCOR, diagnoses are coded using the EPCC(1). In patients with multiple CHDs, the most severe defect is coded as the main CHD, according to a consensus-based classification(2).

Abbreviations: ASD, atrial septal defect; AVSD, atrioventricular septal defect; CHD, congenital heart defect; EPCC, European Paediatric Cardiac Code; IVS, intact ventricular septum; LA, left atrial; LV, left ventricle; LVOT, left-ventricular outflow tract; MAPCA, major aortopulmonary collateral artery; MPA, main pulmonary artery; PDA, patent ductus arteriosus; RA, right atrial; RV, right ventricle; RVOT, right-ventricular outflow tract; TGA, transposition of the great arteries; ToF, tetralogy of Fallot; VA, ventriculoarterial; VSD, ventricular septal defect

Supplementary table 3: ATC codes of non-chronic drugs, excluded for sensitivity analysis.

ATC code 1st	2nd	Description	Includes the following (3rd or 4th level of ATC classification)
B	05	Blood substitutes and perfusion solutions	
D	02	Emollients and protectives	
D	08	Antiseptics and disinfectants	
D	09	Medicated dressings	
J	01	Antibacterials for systemic use	
J	07	Vaccines	
P	01	Antiprotozoals	
P	02	Anthelmintics	
P	03	Ectoparasiticides, incl. scabicides, insecticides and repellents	
V	01	Allergens	
V	03	All other therapeutic products	I.a. drugs for treatment of hyperkalemia, hypoglycaemia, antidotes
V	04	Diagnostic agents	
V	06	General nutrients	
V	07	All other non-therapeutic products	I.a. plasters, diluting agents
V	08	Contrast media	
V	09	Diagnostic radiopharmaceuticals	
V	10	Therapeutic radiopharmaceuticals	
V	20	Surgical dressings	

For sensitivity analysis to test the influence of non-chronic drugs on the cumulative definition of polypharmacy, these drug types were excluded.
Abbreviations: ATC, anatomical therapeutic chemical; i.a., among others.

Supplementary table 4: Baseline characteristics.

	**All patients** N=14,138	**Mild CHD** N=8,126	**Moderate CHD** N=4,757	**Severe CHD** N=1,255
Age at inclusion, *years (IQR)*	35 (24-48)	38 (27-51)	34 (23-46)	25 (21-33)
Male	6969 (49)	3748 (46)	24930 (52)	7280 (58)
Congenital heart defects				
Ventricular septal defect	2329 (16)	2306 (28)	23 (0.5)	
Atrial septal defect	2284 (16)	2077 (26)	207 (4)	
Bicuspid aortic valve	1435 (10)	1535 (18)		
Left ventricular outflow tract obstruction	945 (7)	582 (7)	363 (8)	
Right ventricular outflow tract obstruction	1076 (8)	1022 (13)	54 (1)	
Patent ductus arteriosus	337 (2)	337 (4)		
Coarctation of the aorta	1402 (10)		1402 (30)	
Tetralogy of Fallot	1103 (8)		1103 (23)	
Marfan syndrome	530 (4)		530 (11)	
pAVSD	429 (3)		429 (9)	
cAVSD	214 (2)		214 (5)	
Ebstein's anomaly	211 (1)		211 (4)	
Functionally univentricular heart	202 (1)			202 (16)
Transposition of the great arteries	563 (4)			563 (45)
ccTGA	136 (1)			136 (11)
Double outlet right ventricle	119 (1)			119 (10)
Pulmonary atresia	201 (1)			201 (16)
Other	622 (4)	367 (5)	221 (5)	34 (3)

Data are presented as number (%) and median (IQR). Abbreviatons: ACHD, adult congenital heart disease; cAVSD, complete atrioventricular septal defect; ccTGA, congenitally corrected transposition of the great arteries; CHD, congenital heart defect; IQR, interquartile range; pAVSD, partially atrioventricular septal defect.

Supplementary table 5: Most common drugs in patients and referents with polypharmacy.

**ACHD patients with polypharmacy at inclusion n=4,037**		**Matched referents with polypharmacy at inclusion n=18,878**	
**Age at inclusion,** ***years (IQR)***	46 (33-59)		49 (35-62)
**Male sex**	40%		43%
**Top 5 most dispensed drugs**			
1. Systemic antibiotics	64%	1. Systemic antibiotics	55%
2. Antithrombotics	54%	2. Anti-inflammatory and antirheumatic products	47%
3. β-blockers	46%	3. Drugs for acid related disorders	39%
4. RAAS inhibitors	41%	4. Dermatological corticosteroids	33%
5. Drugs for acid related disorders	37%	5. Lipid modifiers	27%

Most common drug classes at year of inclusion of subjects with polypharmacy during year of inclusion.
Abbreviations: ACHD, adult congenital heart disease; RAAS, renin-angiotensin-aldosterone-system.

Supplementary figure 2: Gap statistic analysis for the identification of the optimal number of clusters.

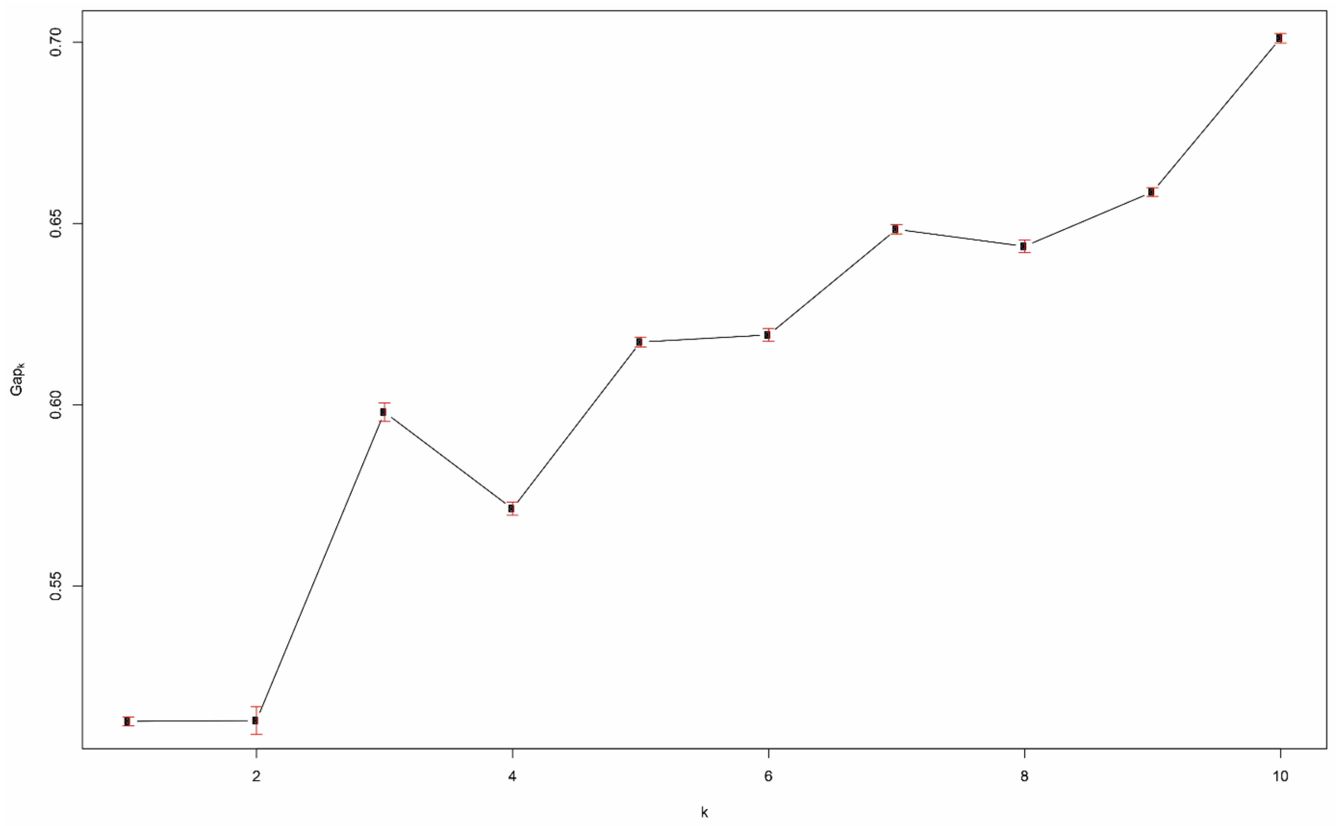

Supplementary table 6: Comparison of medication and clinical characteristics between clusters.

	Cluster 1 *"cardiovascular"* n = 8317 %	Cluster 2 *"low medication use"* n = 3501 %	Cluster 3 *"comorbidity"* n = 2320 %	p-value
**Medication use**				
Medication group[a]				
Alimentary tract	26.7	13.3	23.6	<0.001
Blood & blood forming organs	38.9	10.1	8.6	<0.001
Cardiovascular	50.6	5.6	36.3	<0.001
Dermatologicals	15.5	21.6	49.1	<0.001
Genito-urinary system & sex hormones	10.3	52.6	8.0	<0.001
Systemic hormonal preparations	7.4	4.7	11.7	<0.001
Antiinfectives for systemic use	29.9	58.9	45.6	<0.001
Antineoplastic & immunomodulating agents	1.4	1.4	0.4	<0.001
Musculo-skeletal system	12.0	26.8	38.3	<0.001
Neurological	18.1	15.1	45.3	<0.001
Nervous system	0.7	3.6	0.3	<0.001
Respiratory system	22.3	20.9	31.5	<0.001
Sensory system	12.7	11.3	15.3	<0.001
Amount of medication types				<0.001
0	29.0	0.0	0.0	
1 - 4	40.0	82.3	63.8	
Polypharmacy (≥ 5)	31.0	17.7	36.2	
**Patient characteristics**				
Age, *years (IQR)*	38 (26-52)	28 (22-39)	37 (25-48)	<0.001
Male	57.1	29.8	50.6	<0.001
CHD severity				
Mild	55.8	60.7	58.5	<0.001
Moderate	34.6	30.9	34.5	<0.001
Severe	9.6	8.5	7.0	<0.001
Main diagnosis (most common)				
Ventricular septal defect	14.5	20.4	17.6	<0.001
Atrial septal defect	17.2	14.1	15.6	<0.001
Bicuspid aortic valve	11.2	7.9	9.9	<0.001
Coarctation of the aorta	10.4	8.8	10.1	0.030
Tetralogy of Fallot	7.5	7.8	8.8	0.15
Right ventricular outflow tract obstruction	6.0	10.8	8.5	<0.001
Left ventricular outflow tract obstruction	6.7	7.2	5.9	0.14
Transposition of the great arteries	4.1	4.1	3.5	0.42
Marfan syndrome	4.7	1.3	4.0	<0.001
Partial atrioventricular septal defect	3.0	3.4	2.6	0.23
Patent ductus arteriosus	2.1	2.6	3.0	0.031
Complete atrioventricular septal defect	1.3	2.0	1.4	0.025
Ebstein's anomaly	1.6	1.3	1.5	0.40
Functionally univentricular heart	1.8	1.0	0.9	<0.001
Genetic syndrome with cardiac involvement[b]	5.1	6.3	6.6	0.003
Down's syndrome	2.8	2.7	4.1	0.003

[a]: Medication groups are stated per cluster at the anatomical level of the Anatomical Therapeutic Chemical classification (Supplementary table 1). [b]: excluding Marfan syndrome.

REFERENCES

1. Franklin RC, Anderson RH, Daniels O, Elliott MJ, Gewillig MH, Ghisla R, et al. Report of the Coding Committee of the Association for European Paediatric Cardiology. Cardiology in the young. 2002;12(6):611-8.

2. Warnes CA, Liberthson R, Danielson GK, Dore A, Harris L, Hoffman JI, et al. Task force 1: the changing profile of congenital heart disease in adult life. Journal of the American College of Cardiology. 2001;37(5):1170-5.
